# Optical Coherence Tomography Angiography for the Evaluation of Retinal Vasculature in Fabry Disease: Our Experience and Review of Current Knowledge

**DOI:** 10.3389/fneur.2021.640719

**Published:** 2021-03-09

**Authors:** Daniela Bacherini, Giulio Vicini, Cristina Nicolosi, Ilaria Tanini, Chiara Lenzetti, Lucia Finocchio, Lino Calogero Cirami, Egrina Dervishi, Stanislao Rizzo, Gianni Virgili, Fabrizio Giansanti, Andrea Sodi

**Affiliations:** ^1^Eye Clinic, Neuromuscular and Sense Organs Department, Careggi University Hospital, Florence, Italy; ^2^Cardiomyopathy Unit, Department of Cardiology, Careggi University Hospital, Florence, Italy; ^3^Nephrology Dialysis Transplant Unit, Medical Geriatric Department, Careggi University Hospital, Florence, Italy; ^4^Ophthalmology Unit, Catholic University of the Sacred Heart, Fondazione Policlinico Universitario A. Gemelli, Rome, Italy; ^5^Consiglio Nazionale della Ricerca (CNR), Pisa, Italy; ^6^Department of Neurosciences, Psychology, Drug Research and Child Health, University of Florence, Florence, Italy

**Keywords:** Fabry disease, OCTA, vascular density, vascular perfusion, optical coherenc tomography angiography

## Abstract

**Purpose:** Optical coherence tomography angiography (OCTA) is a non-invasive and objective tool for the evaluation of the retinal microvascular changes in Fabry disease (FD). We investigated changes in retinal vasculature in FD patients, and the possible correlation with systemic parameters, by using OCTA, and reviewed the current status of literature.

**Methods:** Thirteen FD patients (eight females, five males, mean age 49.85 ± 14.7 years) were compared with 13 age- and sex-matched healthy controls. OCTA 3 × 3 mm macular scans were performed in all subjects. We evaluated the vessel density and vessel perfusion in distinct macular areas (whole, inner, and outer) of both the superficial capillary plexus (SCP VD and SCP VP) and of the deep capillary plexus (DCP VD and DCP VP). We also evaluated the foveal avascular zone (FAZ) metrics (area, perimeter, and circularity), and correlation between systemic and OCTA parameters. A literature review on the current understanding of OCTA in FD is then presented.

**Results:** FD patients showed significantly lower SCP VD values in the whole area (17.37 ± 2.08 mm^−1^ vs. 18.54 ± 1.21 mm^−1^; *p*-value 0.022), as well as in the outer area (17.46 ± 2.10 mm^−1^ vs. 19.08 ± 1.14 mm^−1^; *p*-value 0.002), but not in the inner. Even the DCP VD was significantly lower in all the imaged areas: whole (17.75 ± 3.93 mm^−1^ vs. 19.71 ± 1.20 mm^−1^; *p*-value 0.024), outer (18.25 ± 4.17 mm^−1^ vs. 20.33 ± 1.20 mm^−1^; *p*-value 0.023), and inner (19.54 ± 4.17 mm^−1^ vs. 21.96 ± 1.55 mm^−1^; *p*-value 0.011). There were no significant differences in vessel perfusion parameters (both SCP VP and DCP VP ones) and FAZ. No significant correlations were found between the OCTA parameters and systemic parameters (maximal left ventricular wall thickness and glomerular filtration rate) in FD patients.

**Conclusions:** OCTA can be considered as a promising non-invasive tool, which enables a quantitative evaluation of retinal vascular involvement in FD, despite the varying data reported in literature. Our results support the use of OCTA as an objective tool to evaluate retinal vascular abnormalities in FD. The utility of OCTA in FD needs to be validated by longitudinal studies taking into account the overall progression of the disease.

## Introduction

Fabry disease (FD) is a metabolic pathology caused by mutations in the alpha-galactosidase A (α-GAL) lysosomal enzyme gene located on the X chromosome (1). Even if most affected males develop the “classic” phenotype with main involvement of the heart, kidneys, and central and peripheral nervous systems, often with typical skin manifestations, the denomination of “cardiac variants” (2, 3) and “renal variants” (4) have been introduced for patients with predominant or exclusive organ involvement.

Heterozygous females may be asymptomatic carriers even if they may sometimes show a severe phenotype. An increased knowledge of the disease course suggests to define FD as a pathology with a broad spectrum of phenotypes. This spectrum extends from the severe, classic, male phenotype to the apparently asymptomatic form, that can be found in females, with a wide range of intermediate forms of pathology. FD incidence ranges from 1:476,000 (5) to 1:117,000 (6) in the general population even if the disease is commonly considered underdiagnosed. An early FD diagnosis based on the prompt identification of signs and symptoms is crucial to start an effective enzyme replacement therapy. However, the identification of early clinical signs can be difficult due to the heterogeneous presentation, symptoms overlapping with other more common diseases, and less severe kidney or heart involvement in pediatric patients. Recent data indicate that diagnosis occurs on average ~15 years after the first signs or symptoms in both sexes (7).

FD neurological manifestations comprise both peripheral and central nervous system involvement, due to the deposition of globotriaosylceramide in glial and neuronal cells. Cerebrovasculopathy and increased incidence of stroke is the main cause of central nervous system involvement (8). Neuropathic pain, reduced cold and warm sensation, and gastrointestinal disturbances are the main manifestations of peripheral neuropathy in FD. Studies on imaging of cerebral perfusion, cerebral blood flow velocity, and cerebrovascular reactivity showed dysfunction of the cerebrovascular circulation. These studies reported significant cerebral hyperperfusion in patients with FD compared with healthy controls, mainly in the posterior cerebral circulation. Cerebral hyperperfusion appeared to be a vascular phenomenon, related to abnormalities in vasoreactivity and autoregulation, and not caused by neuronal overactivity.

Ophthalmic manifestations of FD include corneal deposits (cornea verticillata), lens opacities, and conjunctival and retinal vessel alterations. Retinal vessel abnormalities mainly consist of increased vessel tortuosity, segmental venous dilation, arteriolar narrowing, and localized vessel constriction. Vascular tortuosity is secondary to the glycosphingolipid deposition in the vessel walls determining a reduced and irregular wall resistance to blood pressure (9, 10).

There are increasing evidences that the retina can reflect cerebrovascular and systemic vascular alterations (11–13). The retinal and cerebral microvasculature share physiological and morphological characteristics; therefore, the clinical assessment of retinal vessels could be a potential marker of the state of the cerebrovasculature.

Traditionally, retinal vessel tortuosity could be remarked by ophthalmoscopy and is best appreciated with fluorescein angiography. However, the introduction of optical coherence tomography angiography (OCTA) offers a more efficient, non-invasive tool for retinal vasculature evaluation. The lack of reliable and universally accepted biomarkers for FD diagnosis and monitoring disease progression has stimulated the study of the ocular manifestations of the disease, in order to find objective and easily detectable parameters, as retinal vessel diameter and tortuosity. In particular, different studies have recently assessed the role of OCTA in the evaluation of the retinal microvascular changes in FD, suggesting that it can be considered an objective and non-invasive method, despite reporting conflicting results (14–20).

In the present study, we conducted an analysis on OCTA results obtained in a group of FD patients regularly followed in the FD Referring Center of Careggi University Hospital in Florence. We evaluated with OCTA the quantitative and qualitative changes in retinal vasculature and the possible correlation with systemic parameters. We also considered a new retinal vasculature parameter, vascular perfusion, never previously investigated in this kind of patients.

## Materials and Methods

This observational case-control study enrolled 13 unrelated patients with FD (FD group) and a control group of 13 healthy individuals without any significant ocular pathology. The study was approved by the Careggi University Hospital Research Ethics Board and adhered to the tenets of the declaration of Helsinki. All patients signed a written consent agreeing to participate. The diagnosis of FD was based on the typical manifestations of the disease, the demonstration of a deficiency of α-GAL in plasma and leukocytes, and the family history. The diagnosis was confirmed by a molecular genetic analysis of α-GAL gene that identified a pathogenic mutation in all the patients. Clinical and imaging data from patients with genetically proven FD were reviewed.

The control group consisted of some members from the medical and nursing staff at the Eye Clinic of the Careggi University Hospital in Florence who voluntarily joined the study.

Patients with myopia > 6D, astigmatism > 3D, relevant ocular pathologies other than FD-related alterations, or optical opacities that precluded good visualization of the fundus were excluded. Other exclusion criteria were smoking (over 10 cigarettes/day), significant systemic diseases other than FD, with particular attention to cardiovascular disorders, and use of medications affecting the circulatory system.

Signs of systemic involvement (neurological, cardiovascular, and renal) were recorded for all the patients included in the study. Some parameters have been considered representative of systemic involvement in FD patients, such as maximal left ventricular wall thickness (MLVWT mm) and glomerular filtration rate (GFR ml/min/1.73 mq). The estimated glomerular filtration rate was determined by the four-variable Modification of Diet in Renal Disease (MDRD) equation, and the interventricular septum diameter was assessed by echocardiography.

Patients included in the study underwent a complete ophthalmic examination including best-corrected visual acuity (BCVA) measurement, intraocular pressure evaluation, slit lamp examination of the anterior segment and dilated fundus examination, and traditional B-scan OCT and OCTA.

The presence of conjunctival or retinal vessel tortuosity, cornea verticillata, or cataract was evaluated by specifically trained ophthalmologists (AS and DB).

OCT and OCTA were obtained with the RS-3000 Advance 2 OCT (NIDEK Co. Ltd., Gamagori, Japan). OCTA RS-3000 Advance 2 uses an 880-nm wavelength with a scanning speed of 85,000 A scans/s and provides high-quality images that enable the qualitative and quantitative assessments required for this study. OCTA examination was performed under full pharmacologic mydriasis with topical tropicamide 1%. OCTA images at 3 × 3 mm were used for all analyses. Vascular retinal layers were visualized and segmented in the SCP and DCP. OCTA scans were reviewed by two investigators (AS and DB) for segmentation accuracy. Automated segmentation of SCP and DCP was performed. Manual adjustment of the segmentation was performed in the presence of cytoarchitectural alterations of the macula. We conducted a qualitative and quantitative assessment.

**Figure 1:**
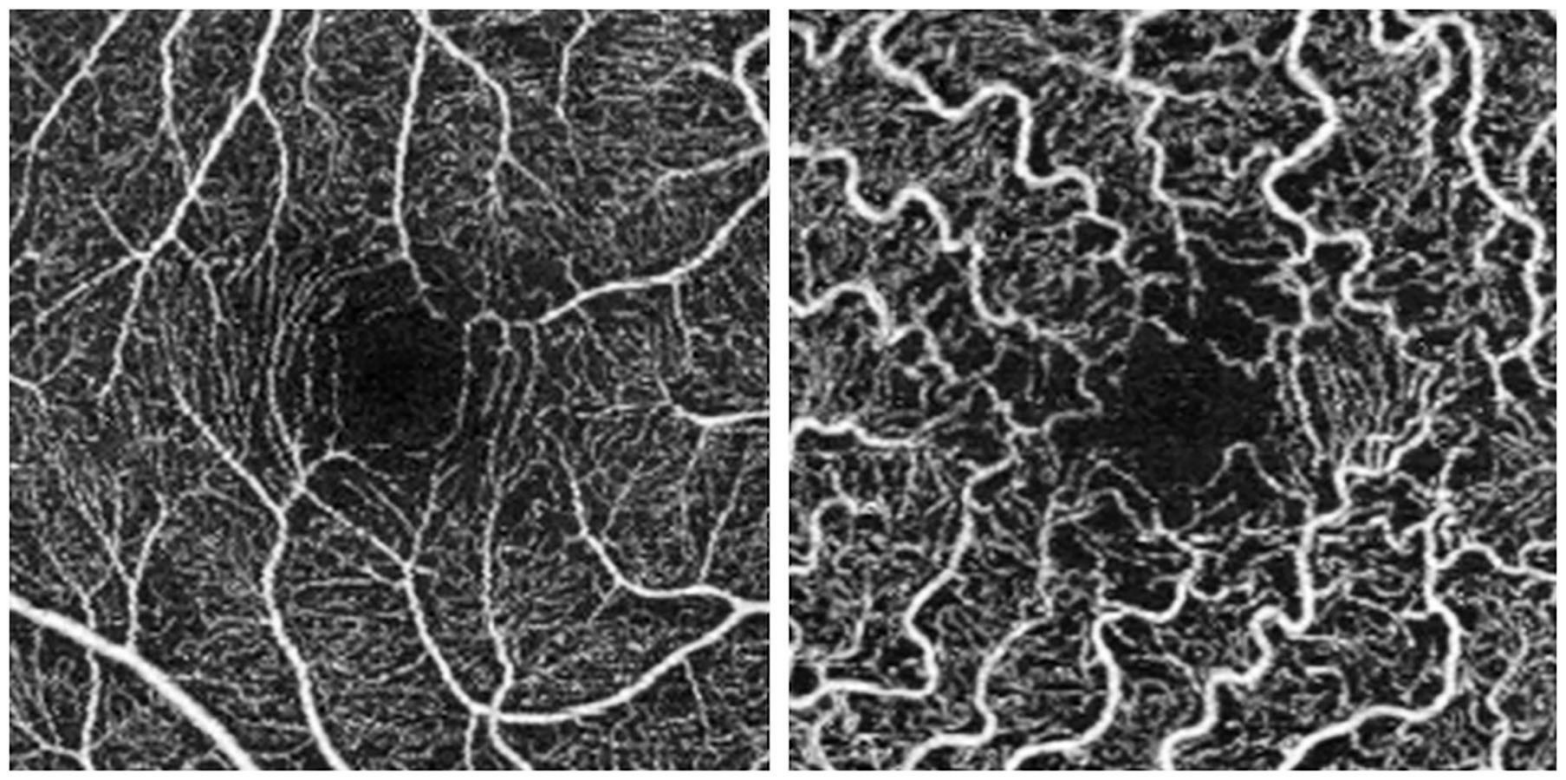
Optical coherence tomography angiography (OCTA) 3 × 3 scans. Scan of the superficial capillary plexus in a healthy control (on the left) and in a patient with Fabry disease (on the right).

Figure 1 shows OCTA scans of SCP in a healthy control and in a patient with Fabry disease. We included in the study only high-quality scans, with a mean SSI 9.4/10. We excluded poor-quality scans, and scans with incorrect segmentation or motion artifacts.

**Figure 2:**
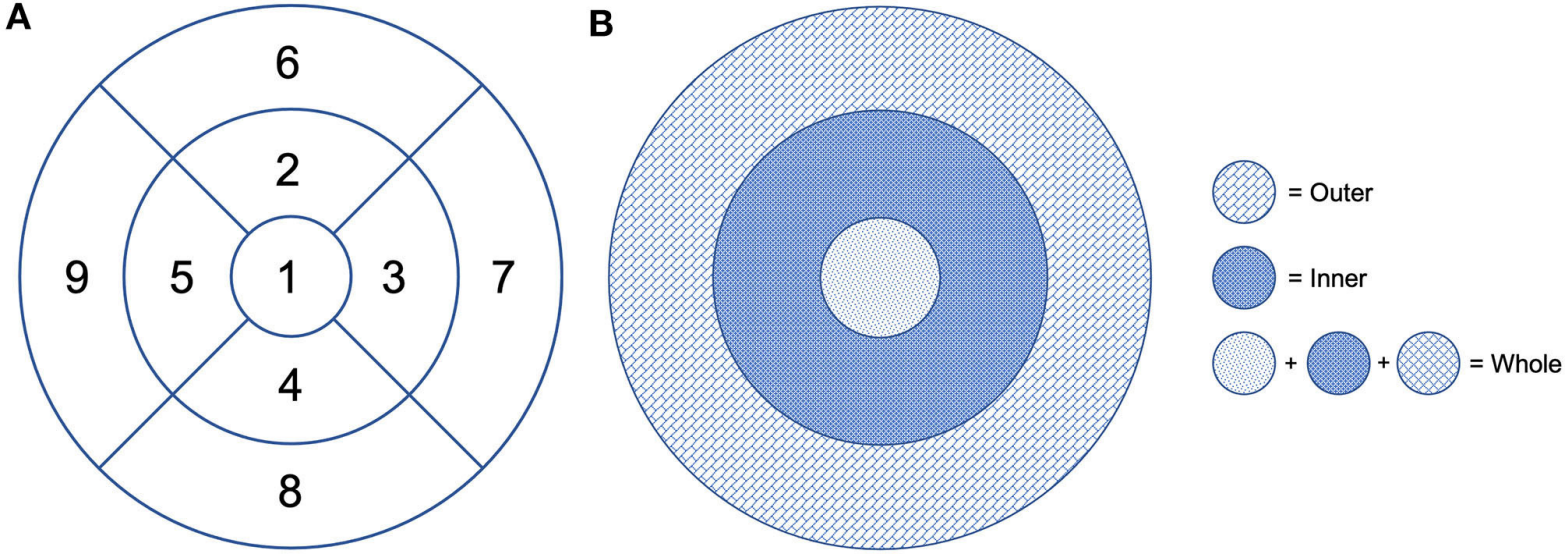
Early Treatment Diabetic Retinopathy (ETDRS) grid centered on the fovea. **(A)** Division of the macular area into nine subfields: 1 = center, 2 = inner superior, 3 = inner right, 4 = inner inferior, 5 = inner left, 6 = outer superior, 7 = outer right, 8 = outer inferior, and 9 = outer left. **(B)** The fovea is defined as the area within the central 1-mm ring of the ETDRS grid. The surrounding ring with an inner diameter of 1 mm and an outer diameter of 3 mm is considered as the inner ring. The ring with an inner diameter of 3 mm and an outer diameter of 6 mm is considered as the outer ring. The whole includes the fovea and the inner and outer rings.

The default RS-3000 Advance 2 OCTA AngioScan software was used to evaluate the vessel density [Early Treatment Diabetic Retinopathy (ETDRS)-based vessel density (mm^−1^)] of the SCP (SCP VD) and DCP (DCP VD) and the vessel perfusion [ETDRS-based vessel perfusion (%)] of the SCP (SCP VP) and the DCP (DCP VP), according to the nine ETDRS subfields (Figure 2A). Vessel density was defined as the absolute length of the perfused vasculature per area in a considered region of estimation. Its units are mm/mm^2^ ranging from 0 (no vessels) to an unbounded value. Vessel perfusion is characterized as the complete zone of perfused vasculature per unit of the considered area. It is expressed as a percentage extending from 0% (no perfusion) to 100% (completely perfused).

Vessel density and perfusion of the SCP and DCP were automatically calculated by the software on OCTA 3 × 3-mm volume scans in the whole foveal, and inner and outer retinal area (Figure 2B).

With the AngioScan software, the angiography image can be converted into two color-coded types of vascular density maps, differently calculated. The vessel density map displays the total linear millimeter of vessels per square millimeter regardless of the vessels original thickness. The perfusion density map considers the original thickness of the vessels when calculating density to display the percentage of tissue that is perfused by blood flow. The AngioScan software automatically detects the foveal avascular zone (FAZ) on SCP and provides quantitative metrics, including its area (mm^2^), perimeter (mm), and circularity.

Statistical analysis was performed using IBM SPSS Statistics (IBM Corporation, Somers, NY) software for Mac (version 26.0). Descriptive statistics such as average values and standard deviations were calculated for all quantitative variables.

Normal distribution of the data was determined by the Shapiro–Wilk test. Between-group comparisons were performed using a two-tailed Student's *t*-test with 95% confidence intervals. Correlations between systemic parameters (maximal left ventricular wall thickness and glomerular filtration rate) and OCTA vascular parameters (SCP VD, DCP VD, SCP VP, DCP VP, FAZ area, FAZ perimeter, and FAZ circularity) in the FD group were calculated using Pearson's correlation test. Statistical significance was defined as a *p* < 0.05.

## Results

In our work, we considered a group of FD patients who underwent a comprehensive ophthalmological examination including OCTA of the posterior pole. Their OCTA data were compared with those obtained with the same procedure in a sex- and age-matched control group.

### Fabry Disease Group

The study included 13 patients (26 eyes) with a certain diagnosis of FD. The mean age of FD patients was 49.85 years (± 14.7, range 22–72), five patients (38.5%) were male, and eight (61.5%) were female. The mean BCVA was 0.10 LogMAR (± 0.05). Eleven patients had good visual acuity bilaterally (0 LogMAR), and a few patients had slightly decreased vision due to mild cataract. Mean intraocular pressure was 13.0 mmHg. Ten out of 13 (76.9%) FD patients had cornea verticillata. Three out of 13 (23%) patients had cataract; two had bilateral lens opacities, while the other patient showed a unilateral cataract in the right eye. Retinal vessel tortuosity in at least one eye was observed in 5 out of 13 (38.5%) patients. Conjunctival vessel tortuosity was present in 8 out of 13 (61%) patients. Traditional macular OCT scans showed no alterations in retinal thickness, morphology, and reflectivity in all cases.

Ten patients (76.9%) in the FD group had systemic involvement, including sensory peripheral neuropathy (mild and not in therapy) (*n* = 3, 23.1%), cardiac involvement (*n* = 7, 53.8%) with slight heart defects detected on echocardiography or electrocardiography (left ventricular hypertrophy, valve failure with mild aortic or mitral regurgitation, and/or conduction abnormalities), chronic kidney disease (*n* = 6, 46.15%), defined as GFR <60 ml/min/1.73 mq, proteinuria (>150 mg/24 h), urine sediment abnormalities, and/or electrolyte abnormalities. One patient had undergone kidney transplantation and was on dialysis during this study. Six patients (*n* = 6, 46.15%) were in NYHA class II. The average MLVWT was 11.9 mm. The mean GFR value was 87.3 ml/min/1.73 mq.

Nine out of 13 (69.2%) patients were receiving treatment at the time of the study: 12 patients were receiving enzyme replacement therapy (ERT) and one patient, previously treated with ERT, was receiving chaperone therapy with Migalastat.

Table 1 summarized the main demographics and clinical features of FD patients included in our series.

**Table 1:** Demographics and clinical data of the patients with Fabry disease.

	**Age**	**Sex**	**Organ involvement**	**NYHA**	**MLVWT**	**GFR**	**Cornea verticillata**	**Retinal vessels tortuosity**	**Mutation**	**Therapy**
1	64	F	B,H	I	14	82	Yes	No	c.902G>C p.(Arg301 Pro)	ERT/PC
2	65	M	H,K	II	21	78	No	No	c.644A>G p.(Asn215Ser)	ERT
3	46	F	None	I	6	100	No	No	c.728T>C p.(Leu243Ser)	No
4	42	F	None	I	8	90	No	No	c.427G>A p.(Ala143Thr	No
5	72	F	B,H	II	14	76	Yes	No	c.902G>C p.(Arg301 Pro)	ERT
6	62	F	H	II	14	100	Yes	Yes	c.1091_1092del p.(Ser364Leufs^*^10)	ERT
7	47	F	B,H,K	II	10	75	Yes	No	c.902G>C p.(Arg301 Pro)	No
8	31	F	B	I	8	150	Yes	No	c.1091_1092del p.(Ser364Leufs^*^10)	ERT
9	22	F	None	I	8	100	Yes	No	c.902G>C p.(Arg301 Pro)	No
10	53	M	H,K	II	10	31	Yes	Yes	c.188G>A (p.Cys63Tyr)	ERT
11	60	M	B,H,K	II	19	50	Yes	Yes	c.334C>Tp.(Arg112Cys)	ERT
12	36	M	K	I	12	130	Yes	Yes	c.1091_1092del p.(Ser364Leufs^*^10)	ERT
13	48	M	B,K	I	11	3	Yes	Yes	c.902G>C p.(Arg301 Pro)	ERT

*B, brain/neuropathy; H, heart; K, kidney; NYHA, New York Heart Association; MLVWT, maximal left ventricular wall thickness (mm); GFR, glomerular filtration rate (ml/min/1.73 mq); ERT, enzyme replacement therapy; PC, pharmacological chaperone*.

### Control Group

The control group consisted of 13 healthy individuals (26 eyes). The mean age was 50 years (± 15.2, range 26–73), five patients (38.5%) were male, and eight (61.5%) were female. These patients had no history of significant ocular and systemic pathology. BCVA was 0 logMar in all cases. On average, mean intraocular pressure was 14 mmHg. Anterior segment and dilated fundus examination were unremarkable in both eyes of all patients. No alterations in macular thickness, morphology, and reflectivity were observed on traditional OCT scans.

### Optical Coherence Tomography Angiography Results

**Figure 3:**
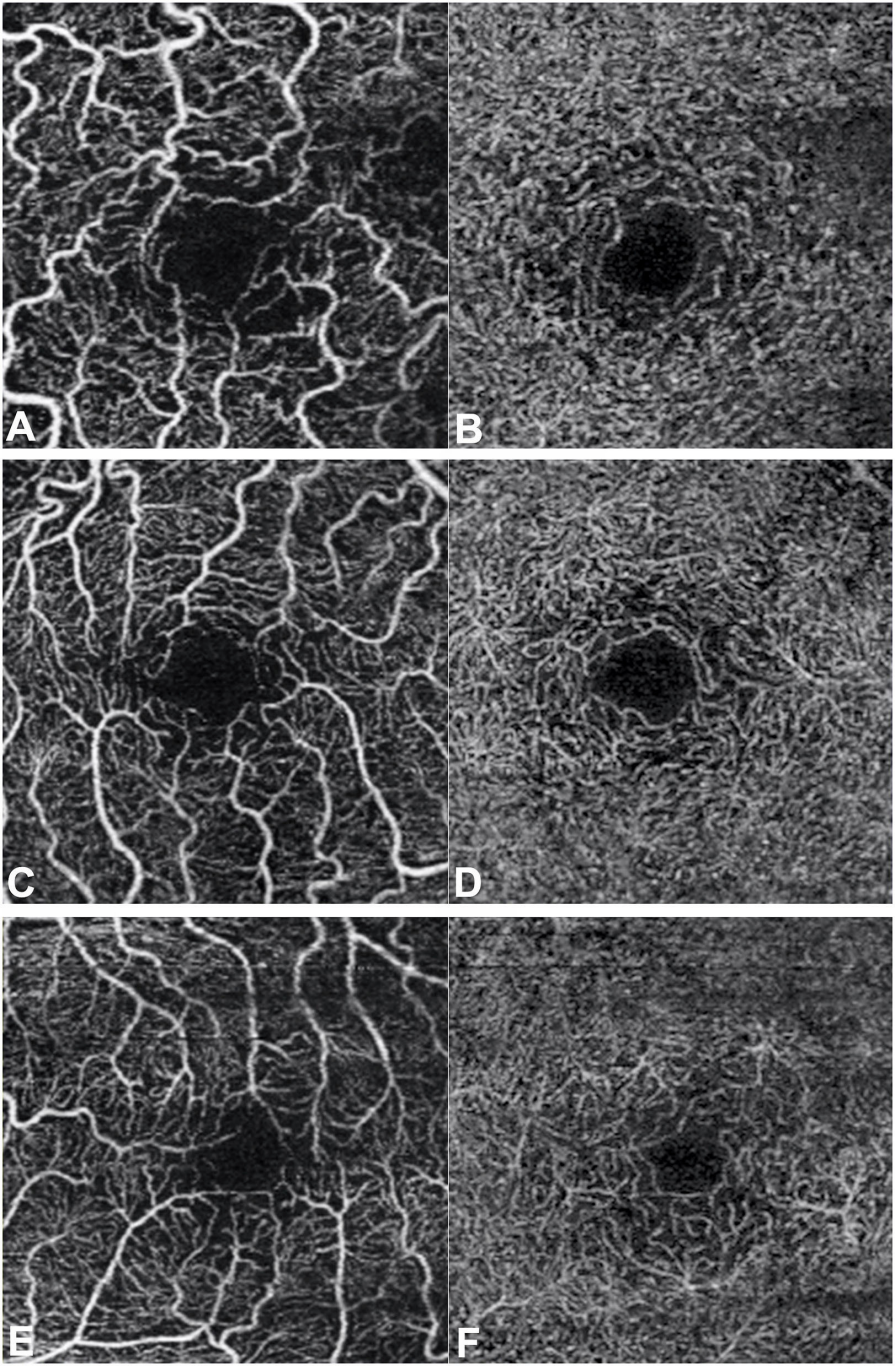
Optical coherence tomography angiography scans (3 × 3 mm) in patients with Fabry disease. Rarefaction of the vascular texture of both the superficial capillary plexus **(A,C,E)** and the deep capillary plexus **(B,D,F)** can be observed.

**Figure 4:**
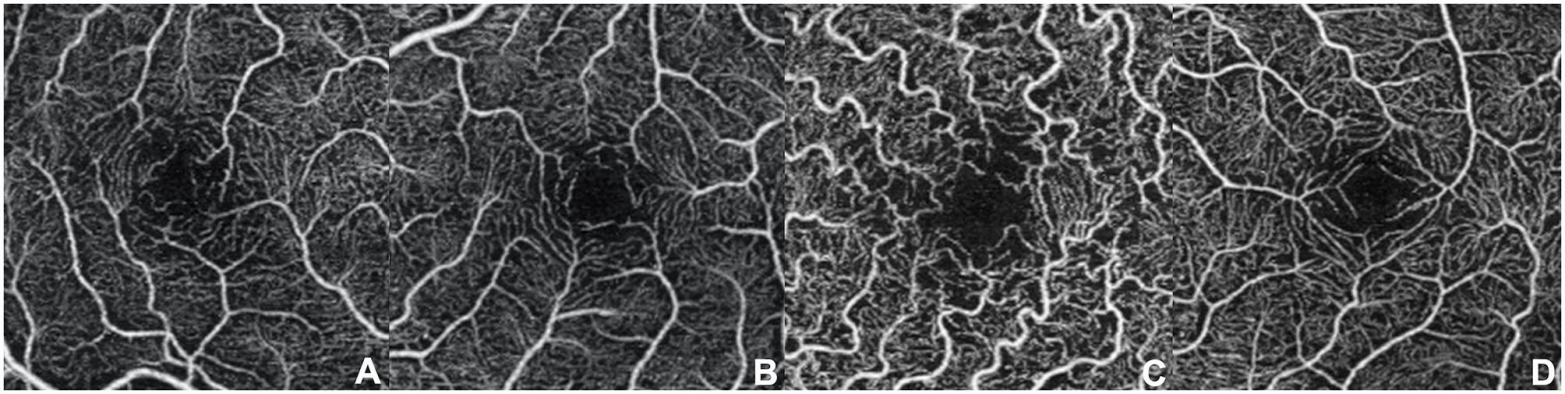
Optical coherence tomography angiography scans (3 × 3 mm) at the superficial capillary plexus in patients with Fabry disease. **(A–D)** Morphological alterations of the foveal avascular zone with irregular borders and sudden changes in the direction of the vessels with perifoveal loops can be observed.

**Figure 5:**
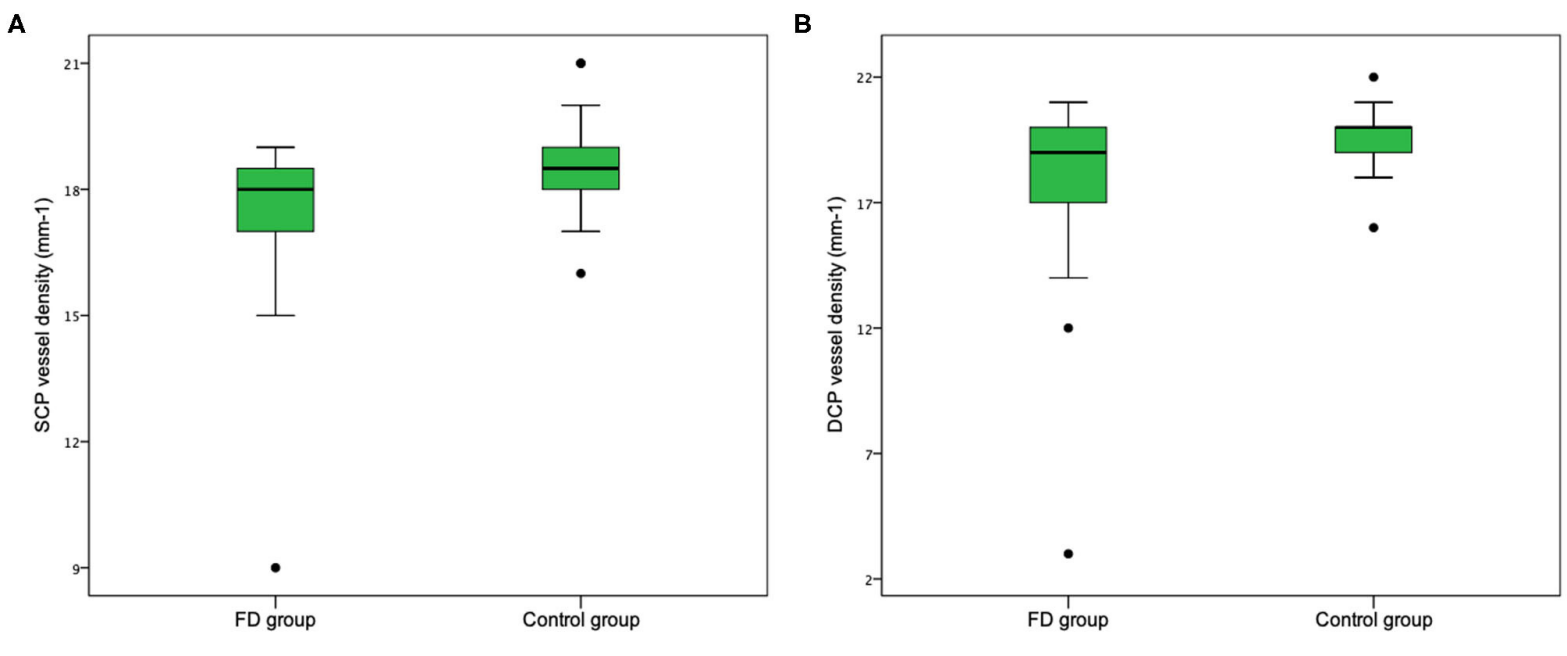
Retinal vessel density in patients with Fabry disease and normal control group. Vessel density in both the whole superficial capillary plexus (SCP) **(A)** and the whole deep capillary plexus (DCP) **(B)** is lower in the group of patients affected by Fabry disease.

Even if our study mainly focused on quantitative vascular abnormalities, a few qualitative features could be reported in FD patients. Specifically, we could remark in FD a rarefaction of the vascular texture both in the SCP and in the DCP (Figure 3). Additionally, the FAZ of the FD group often had irregular borders and sudden changes in the direction of the vessels with perifoveal loops (Figure 4). Then considering the quantitative results, the FD group showed, in comparison with normal subjects, significantly lower SCP VD values in the whole area (17.37 ± 2.08 mm^−1^ vs. 18.54 ± 1.21 mm^−1^; *p*-value 0.022) (Figure 5A), as well as in the outer area (17.46 ± 2.10 mm^−1^ vs. 19.08 ± 1.14 mm^−1^; *p*-value 0.002), but not in the inner (19.33 ± 2.22 mm^−1^ vs. 20.37 ± 1.55 mm^−1^; *p*-value 0.066). Even the DCP VD was significantly lower in the FD group: in particular, we observed a significant reduction in all the imaged areas, whole (17.75 ± 3.93 mm^−1^ vs. 19.71 ± 1.20 mm^−1^; *p*-value 0.024) (Figure 5B), outer (18.25 ± 4.17 mm^−1^ vs. 20.33 ± 1.20 mm^−1^; *p*-value 0.023), and inner (19.54 ± 4.17 mm^−1^ vs. 21.96 ± 1.55 mm^−1^; *p*-value 0.011). There were no significant differences in vessel perfusion parameters (both SCP VP and DCP VP ones) and FAZ parameters (area, perimeter, and circularity) between the two study groups. A complete list of the comparisons in OCTA parameters between controls and FD patients are reported in Table 2.

**Table 2:** Differences in OCTA parameters between patients with Fabry disease and healthy controls.

**OCTA parameter**	**FD group**	**Control group**	*p*-value^*^
**SCP VD (mm**^**–1**^**)**			
Whole	17.37 ± 2.08	18.54 ± 1.21	**0.022**
Outer	17.46 ± 2.10	19.08 ± 1.14	**0.002**
Inner	19.33 ± 2.22	20.37 ± 1.55	0.066
**DCP VD (mm**^**–1**^**)**			
Whole	17.75 ± 3.93	19.71 ± 1.20	**0.024**
Outer	18.25 ± 4.17	20.33 ± 1.20	**0.023**
Inner	19.54 ± 4.17	21.96 ± 1.55	**0.011**
**SCP VP (%)**			
Whole	50.62 ± 5.45	51.37 ± 1.53	0.520
Outer	52.04 ± 6.07	53.87 ± 1.23	0.154
Inner	55.17 ± 5.58	55.00 ± 2.50	0.686
**DCP VP (%)**			
Whole	44.37 ± 9.91	47.83 ± 3.16	0.110
Outer	47.33 ± 10.69	51.58 ± 3.19	0.068
Inner	46.71 ± 10.04	50.71 ± 3.38	0.071
FAZ area (mm^2^)	0.26 ± 0.11	0.28 ± 0.09	0.624
FAZ perimeter (mm)	2.59 ± 0.71	2.56 ± 0.60	0.872
FAZ circularity index	0.48 ± 0.09	0.53 ± 0.10	0.060

*Data presented as mean ± SD*.

* *Student's t-test, statistical significance was defined as p < 0.05; SCP VD, vessel density of superficial capillary plexus; DCP VD, vessel density of deep capillary plexus; SCP VP, vessel perfusion of superficial capillary plexus; DCP VP, vessel perfusion of deep capillary plexus; FAZ, foveal avascular zone. The bold values are that statistically significant (p < 0.05)*.

In our series, we did not find any significant correlation between the OCTA parameters and systemic parameters (MLVWT and GFR) in FD patients. A complete list of the results of this analysis are reported in Table 3.

**Table 3:** Correlation between OCTA parameters and systemic parameters in patients with Fabry disease.

**OCTA parameter**	**MLVWT**		**GFR**	
	***r***	***p*-value**	***r***	***p*-value**
SCP VD whole	−0.18	0.41	0.33	0.12
SCP VD inner	−0.20	0.36	0.36	0.08
SCP VD outer	−0.65	0.76	0.27	0.20
DCP VD whole	−0.14	0.51	0.12	0.58
DCP VD inner	−0.21	0.32	0.20	0.33
DCP VD outer	−0.12	0.59	0.11	0.62
SCP VP whole	−0.13	0.53	0.8	0.69
SCP VP inner	−0.14	0.52	0.72	0.74
SCP VP outer	−0.14	0.50	0.17	0.42
DCP VP whole	−0.11	0.66	0.06	0.78
DCP VP inner	−0.13	0.55	0.08	0.72
DCP VP outer	−0.09	0.66	0.04	0.85
FAZ area	−0.43	0.84	0.20	0.34
FAZ perimeter	0.68	0.75	−0.06	0.78
FAZ circularity	−0.21	0.33	0.38	0.68

*r, Pearson's correlation coefficient; SCP VD, vessel density of superficial capillary plexus; DCP VD, vessel density of deep capillary plexus; SCP VP, vessel perfusion of superficial capillary plexus; DCP VP, vessel perfusion of deep capillary plexus; FAZ, foveal avascular zone area; MLVWT, maximal left ventricular wall thickness; GFR, glomerular filtration rate*.

## Discussion

In FD, the progressive deposition of non-catabolized glycosphingolipids in endothelial cells, smooth muscle, and pericytes causes vessel wall thickening and lumen narrowing, with consequent local tissue ischemia (21–23).

These vascular alterations in association with the increased platelet activity and inflammation may lead to the development of stroke, coronary dysfunction, and renal failure, with reduced life expectancy (24). Retinal vessel abnormalities in FD are mainly represented by increased tortuosity, sometimes with a “corkscrew” appearance, segmental venous dilation, arteriolar narrowing, and arteriovenous nicking (localized constriction) (25–28). Retinal vascular alterations may be related to retinal ischemia and its complications (25, 29, 30).

There is still a lack of objective and reproducible methods to assess the retinal vascular changes in FD patients, in order to allow early diagnosis of the disease, provide a quantitative evaluation of vascular involvement, and follow its progression over time. In fact, previous mere descriptive analyses of the posterior segment in FD patients were subjective and poorly reproducible (31). Computer-assisted analysis of retinal vessel tortuosity and diameter, evaluated from fundus images by means of a dedicated software, showed more tortuous and narrower retinal arteries in FD patients than in controls, providing an objective and non-invasive tool to evaluate vascular alterations in FD (31–34). Recently, adaptive optics ophthalmoscopy of retinal vessels in FD patients allowed the detection of vascular deposits, which may be useful for a gradable evaluation of microvascular involvement (35).

OCTA is an objective and non-invasive tool for the evaluation of the retinal microvascular changes in FD, even if different studies report varying and not conclusive data (14–20). Table 4 summarizes the results of these studies.

**Table 4:** Studies reported in literature regarding optical coherence tomography angiography in patients affected by Fabry disease.

**Study authors**	**Number of FD patients/controls**	**OCTA device**	**Scan width (mm)**	**Findings in Fabry disease group**
Finocchio et al. (14)	13/13	Spectral domain	3 × 3	Reduction in DCP vascular density, without differences in SCP. Reduction in FAZ area of SCP and DCP
Hufendiek et al. (15)	10/10	Spectral domain	3 × 3	Reduction in superficial, deep, and choriocapillaris flow density in all parafoveal sectors (except superficial layer in the central sector)
Baur et al. (16)	14/8	Swept source	3 × 3 and 6 × 6	Reduction in DCP vascular density, without differences in SCP
Cennamo et al. (17)	54/70	Spectral domain	6 × 6	Reduction in vessel density in SCP (whole image, fovea, and parafovea) and increase in the DCP (whole image, fovea, and parafovea)
Minnella et al. (18)	20/17	Swept source	4.5 × 4.5	Increase in SCP vascular density but not in the other layers, and enlargement of FAZ areas both in the SCP and DCP
Cakmak et al. (19)	25/37	Spectral domain	Macular 6 × 6 Disk 4.5 × 4.5	Reduction of vessel density in both foveal SCP and DCP. Enlargement of FAZ area. No differences in density of radial peripapillary capillaries
Dogan et al. (20)	38/40	Spectral domain	6 × 6	Lower DCP vascular density without any changes in SCP and choriocapillaris vascular density

*DCP, deep capillary plexus; SCP, superficial capillary plexus; FAZ, foveal avascular zone*.

In our series we quantitatively assessed with OCTA the retinal vasculature, and we found a statically significant lower vascular density, in both SCP (whole and outer area) and DCP (whole, outer, and inner area), in the FD group compared with those in the controls. On the other side, there were no significant differences in vascular perfusion indices (both for SCP and DCP) and in FAZ parameters (area, perimeter, and circularity).

So far, only a few studies have assessed the retinal vasculature with OCTA in FD, showing discordant results (15–20). In addition, different results emerged from a preliminary study of our group, performed using an earlier version of the OCTA image analysis software (14). In our previous work, we observed a statistically significant reduction in the vascular density values only in DCP, but not in the SCP. In that study also, the FAZ parameters were statistically lower in both the SCP and DCP. Another study conducted by Baur et al. (16), by using swept source OCTA scans (3 × 3 mm and 6 × 6 mm), found a statistically significant reduction only in the DCP vascular density values.

Cennamo et al. (17), using spectral-domain OCTA 6 × 6-mm images, carried out a prospective study in order to evaluate vessel density in different macular areas (whole image, fovea, and parafovea) in FD patients. They found that the vessel density was significantly reduced in the SCP (in each macular area) and significantly increased in the DCP (in each macular area) in FD patients (*n* = 54) compared with the healthy controls (*n* = 70). They attributed the decrease in the vessel density at the level of the SCP to a reduction in the blood flow, due to vascular narrowing and tortuosity, or to blood flow impairment and hypercoagulability reported in FD. They hypothesized that the increased vessel density in DCP was due to a compensatory vascular mechanism, to balance the reduction of vessel density in the SCP, with the recruitment of microvascular units in DCP.

Minnella et al. (18), using swept source OCTA 4.5 × 4.5-mm scans measured the vascular density score for the various chorio-retinal layers in 20 FD patients (38 eyes). They found in FD patients a significant increase in the vascular density in the SCP but not in the other layers, and a significant enlargement of FAZ areas both in the SCP and DCP. They explained this result apparently in contradiction with the FAZ enlargement and the rarefaction of retinal capillary plexuses, as an effect of an increased vascular tortuosity that may affect OCT signal reflectance, yielding a density score increase. They also used the focal electroretinography (fERG) method to further investigate the macular function and found a decreased fERG amplitude with preserved phase values in FD patients, suggesting a subclinical dysfunction of the outer retinal layers. They suggested that compromised vascular blood supply can harm photoreceptors and bipolar cells. However, no significant correlations were found between FAZ enlargement and fERG values, implying that, in addition to tissue perfusion deficits, other factors might be responsible for the observed abnormalities in outer retinal function.

A study by Cakmak et al. (19), conducted on spectral-domain OCTA 6 × 6-mm images, found significantly lower vessel density in FD patients (*n* = 25) than in the controls (*n* = 37), in both foveal SCP and foveal DCP. The vessel density in FD patients was lower in almost every macular sector, although the differences were found to be not statistically significant except for the fovea. The general reduction of the macular vessel density in FD patients would be a consequence of the characteristic retinal vascular changes. The FAZ was significantly larger in the FD group than in the control group. This study also assessed the density of radial peripapillary capillaries on 4.5 × 4.5 scans focused on the optic disk, and no significant differences were found between the two groups.

Dogan et al. (20), by using spectral-domain OCTA 6 × 6-mm images, found lower DCP vascular density in FD patients (*n* = 38) without any changes in SCP and choriocapillaris compared with the healthy controls (*n* = 40). This decrease was associated mostly with the renal involvement and duration of treatment. They speculated that this finding, in addition to retinal vascular changes (with vascular constriction and increased tortuosity), could be attributable to increased hydrostatic pressure in DCP, secondary to vascular hypercoagulability and abnormal vascular flow, similar to the situation observed in retinal vein occlusion. This increased pressure might lead to decreased perfusion in the related retinal area causing a decrease in the capillary vascular density. However, SCP might have protected itself better compared to the DCP. The absence of differences in choriocapillary blood flow between FD patients and controls might be related to the specialization difference in the choriocapillaris, with a highly polarized appearance. However, FD patients with hypertension had a significantly decreased choriocapillaris blood flow compared with the ones without hypertension, which is consistent with the ischemic choroidal changes and choroidal hypoperfusion in hypertensive choroidopathy.

In our study, we used 3 × 3-mm macular angiography scans, which appear to be more suitable than larger ones (6 × 6-mm scans) for quantitatively analyzing the macular microvascular metrics and the FAZ in terms of repeatability (36). We observed a statistically significant reduction in vascular density indices in both SCP and DCP in FD patients compared with the controls. This result is in agreement with a study by Hufendiek et al. (15) conducted on 3 × 3-mm OCTA images obtained using a spectral-domain OCT, which showed a mean superficial, deep, and choriocapillaris flow density significantly decreased in FD patients compared with the healthy controls.

This disagreement in vascular density values in different retinal layers observed in published studies may reflect a different degree of eye involvement in FD, as a result of different stages of disease at the time of analysis. We should also consider that these studies are performed in most cases on limited numbers of patients (as FD is a rare disease), and moreover, different devices have been used for image acquisition, with different modalities of data analysis.

In the current study, OCTA was used for a quantitative assessment of retinal vasculature in FD patients. The data showed a decrease in vascular density both in the SCP and in the DCP consistent with the reports of retinal vascular abnormalities due to metabolite deposition in FD (26). In our series, there were no differences in OCTA FAZ parameters between the two study groups. Decreased blood flow usually results in an enlarged FAZ due to increased ischemia. We can only conjecture that the irregular borders of the FAZ and the anomalous course of some perifoveal vessels in FD interferes with a reliable automated evaluation of the FAZ area.

To date, the published studies have evaluated only OCTA parameters related to vascular density, but not to perfusion. Our study also investigated vascular perfusion parameters (SCP VP and DCP VP), which, however, did not show statistically significant differences between FD patients and healthy controls. The cause of this results, with a significant reduction in the OCTA indices of vascular density, but without a reduction in the perfusion indices, remains unclear. We can assume the intervention of vascular compensation mechanisms to counteract the reduction of vascular density and the presence of subclinical retinal ischemia. This discrepancy between density and perfusion OCT vascular indices was described in other disorders, such as chronic Leber hereditary optic neuropathy, in which it has been hypothesized to be secondary to a more prominent damage of the small vessels (37).

In our study, we also investigated the correlations between OCTA results and two systemic parameters, indicators of cardiac and renal impairment attributable to FD (MLVWT and GFR): this analysis did not show a statistically significant relationship with these variables.

Not even Minnella et al. (18) found a correlation between their results and the systemic severity of the disease quantified by calculating the Mainz severity score index. Dogan et al. (20) found that FD patients with renal involvement showed a lower DCP vascular density compared with healthy controls, which is consistent with a recent study on patients with chronic kidney disease (38): this result suggests that the reduction in the retinal vascular density might be related to systemic complications such as renal involvement rather than the FD itself. Most of the patients included in our series did not show severe renal involvement, considering also that the loss of filtering capacity in kidney dysfunction may be preceded by a period of abnormally high GFR, or hyperfiltration (39); this likely contributed to the absence of significant correlation between GFR and OCTA parameters.

A recent study by Cennamo et al. (40) evaluated the relationship between the changes in retinal vessel density by OCTA and the vascular alterations involving renal, cardiovascular, and central nervous systems in FD patients: the vessel density of SCP and DCP was inversely related to echocardiographic parameters (early mitral inflow peak velocity to early diastolic mitral annulus peak velocity ratio, left atrial volume index, interventricular septal thickness, global longitudinal strain, and systolic pulmonary artery pressure); however, no relationship was found, with a multivariate analysis, between OCTA parameters and kidney function and neuroradiological signs of central nervous system involvement.

We did not find a significant correlation between OCTA indices and MLVWT, reflecting the degree of left ventricular hypertrophy; however, the coronary microvascular function may be impaired in FD patients irrespective of left ventricular hypertrophy, and it may be an early sign of cardiac involvement, which precedes the development of cardiac hypertrophy (41).

At the present moment, the OCTA data may be potentially useful as a surrogate marker for an indirect investigation of systemic abnormalities in FD, if further studies will confirm it. The investigation of qualitative and quantitative OCTA parameters and the correlations with systemic markers can be considered a newsworthy object of research because of its non-invasiveness, repeatability, and the possibility of *in vivo* evaluation of the parameters.

We are aware that our study presents some potential limitations. The small sample size probably represents the most important drawback. Nevertheless, it should be noted that FD is a rare disorder and that the sample was large enough to allow the detection of statistically significant differences. In our series, the reduced vascular density did not correlate with two parameters of systemic involvement progression (MLVWT and GFR): These parameters are probably not sufficient to demonstrate a significant correlation with OCTA data, and other systemic parameters should be taken into account.

Moreover, the difficulty in distinguishing vascular abnormalities directly attributable to FD from vascular abnormalities related to hypertension may represent another potential limitation as some FD patients develop arterial hypertension during the course of the disease. Recent studies confirmed that systemic hypertension may affect OCTA parameters, being associated with lower values of vascular density and vascular perfusion, and FAZ enlargement (42–45). In our study, primary arterial hypertension detected before the diagnosis of FD was an exclusion criteria, to exclude that vascular impairment may be due to hypertensive retinopathy, but five patients became hypertensive after the diagnosis of FD, as a complication of the disorder. The small size of our series prevented a significant statistical evaluation of possible differences between the subgroup of FD patients who became hypertensive and the remaining FD population. Another limitation is the lack of a reliable measurement of the FAZ. Finally, we must consider that this is a cross-sectional study, which includes a heterogeneous group of FD patients, with different degrees of systemic and ocular involvement, and therefore, it does not allow to evaluate the effects of disease progression over time.

## Conclusions

OCTA is a non-invasive and efficient tool, which enables a good evaluation of the retinal vascular network and provides objective parameters for quantitative assessment of retinal vessels changes in FD. The varying and conflicting data reported in literature can be attributed to several factors, including the different stages of disease, the limited number of patients, the different OCTA devices and data analysis, and therefore require a careful interpretation. Nevertheless, our results support the use of OCTA as an objective tool in the assessment of the retinal vascular abnormalities in FD for an early diagnosis, a quantitative evaluation of vascular involvement. However, our findings and the validity of this imaging modality in evaluating microvascular alterations in FD need to be assessed by further studies, considering also vascular modifications over time, along with the progression of the disease.

## Data Availability Statement

The raw data supporting the conclusions of this article will be made available by the authors, without undue reservation.

## Ethics Statement

The studies involving human participants were reviewed and approved by Comitato Etico Azienda Ospedaliera Universitaria Careggi. The patients/participants provided their written informed consent to participate in this study.

## Author Contributions

DB, GVic, and CN: conceptualization, data curation, writing – original draft preparation, and visualization. LF and CL: data curation and validation. GVir, SR, ED, and LC: supervision and validation. DB, IT, FG, and AS: supervision, validation, writing – review, and editing. All the authors have read and approved the final version of the manuscript.

## Conflict of Interest

The authors declare that the research was conducted in the absence of any commercial or financial relationships that could be construed as a potential conflict of interest.
